# Book Reviews

**Published:** 1916-10

**Authors:** 


### Book Reviews.

PAINLESS CHILDBIRTH. By Carl Henry Davis, A. B., M. D., Associate in Obstetrics and Gynecology, Rush Medical College. Cloth, 134 pp. Chicago, Forbes & Co. Price, $1.00.

Covering the whole subject of eutocia and nitrous oxid-oxygen analgesia, the chemistry, pharmacology and toxicology of various analgesics are discussed in a perfectly fair manner. "Twilight sleep" methods are discussed, but the author favors the newer nitrous oxid-oxygen methods. His technic is the feature of the book.

---

EYE, EAR, NOSE AND THROAT. Practical Medicine Series, 1916. Edited by Casey A. Wood, C. M., M. D., D. C. L.; Albert H. Andrews, M. D.; George E. Shambaugh, M. D. 12mo. 376 pages, $1.50. The Year Book Publishers, Chicago.

The volume of Practical Medicine Series devoted to the eye, ear, nose and throat contains practically everything of value that has been written the past year on ophthalmology, otology, rhinology, and larynxology. One chapter is devoted to eye injuries resulting from high explosives and schrapnel. Attention is given to focal infection, particular tonsilar, dental, intestinal toxemas as they affect the eye. The work is ably edited and is of interest to both specialist and practitioner.

---

VENESECTION. A Brief Summary of the Practical Value of Venesection in Disease. For Students and Practitioners of Medicine. By Walter Forrest Dutton, M. D. Cloth, 220 pp., $2.50. F. A. Davis Co., Philadelphia and London.

Truly there is nothing new under the sun and nothing is lost. After all these years of quietly being ignored by the practitioner "Blood-letting" again claims the attention of the doctor. Venesection is a brief summary of the value of blood-letting in the practice of medicine and the reader will be surprised at the number of diseases in which venesection is recommended as a remedy.

---

CANDY MEDICATION. By Bernard Fantus, M. D., Professor of Pharmacology and Therapeutics, University of Illinois, Chicago. 82 pp. Price, $1.00. C. V. Mosby & Co., St. Louis. 1915.

This book by Fantus is quite unique in that it is the only book of its kind on the market. It evidently was intended primarily for the druggist, because it gives in detail the method of preparation by which a number of drugs can be put in candy form. Formulae and methods of making candy tablets and the drugs which can be so used are given, with full instruction for every detail. Formulae are given for fifty-seven different medications.

---

DISEASES OF THE SKIN. By Richard L. Sutton, M. D., Professor of Diseases of the Skin, University of Kansas School of Medicine, etc. 916 pp., 693 illustrations and eight colored plates. C. V. Mosby & Co., St. Louis. 1916. Price, $6.50.

This volume from Dr. Sutton is well written and well illustrated. The extensive bibliography after each chapter shows a great amount of research work. The volume contains a good description of foot and mouth disease along with several colored prints of this condition. More than the usual amount of space is devoted to pathology and treatment of this disease. The majority of the therapeutic measures advocated by the author are those that he has found useful and practical in his own practice. The book, while comprehensive, is concise and will be a valuable aid in diagnosing and treating skin diseases.

---

Diagnostic Methods. A Guide for History Taking, Making of Routine Physical Examinations, and the Usual Laboratory Tests Necessary for Students in Clinical Pathology, Hospital Internes and practicing Physicians. By Herbert Brooks, A. B., M. D., Professor of Pathology, University of Tennessee. Third Edition, Revised and Rewritten. Cloth, 96 pp., $1.00. C. V. Co., St. Louis, 1916.

This little book is just what it purports to be—a thoroughly reliable, convenient and practical guide for the average practitioner.

---

Diseases of the Digestive Tract and Their Treatment. By A. Everett Austin, A. M., M. D., Former Professor of Physiological Chemistry at Tufts College, University of Virginia and University of Texas; Present Assistant Professor of Clinical Medicine, in Charge of Dietetics and Gastro-intestinal Diseases, Tufts College; Member of American Gastro-enterological Association, and American Society of Biological Chemists; Physician to Mt. Sinai Hospital and Berkeley Infirmary, and Assistant to Boston Dispensary; Author of "Manual of Clinical Chemistry," etc. With eighty-five illustrations, including ten color plates. C. V. Mosby & Co., Publishers, St. Louis, Mo. 1916. Price, $5.50.

In this book Dr. Austin has succeeded in placing on the market a work on diseases of the digestive tract which is not only a source of information, but which is attractively readable to the general practitioner. Dr. Austin presents to the reader a wonderful amount of information. He begins with the anatomy and physiology of the digestive tract, and also takes up its pathology. He also deals with the examination of the patient, physical and chemical methods in gastro-enterology, dietetics, general treatment, and a dozen chapters are devoted to the description of special gastric and intestinal diseases.

The articles on gastric ulcers are especially fine. Those interested in diseases of the intestinal tract will find this book well worth its price.

